# Acquired hemophilia A associated with Epstein–Barr-virus-associated T/natural killer-cell lymphoproliferative disease: A case report

**DOI:** 10.1097/MD.0000000000025518

**Published:** 2021-04-23

**Authors:** Masayo Yamamoto, Motohiro Shindo, Chihiro Sumi, Sho Igarashi, Takeshi Saito, Nodoka Tsukada, Yasumichi Toki, Mayumi Hatayama, Junki Inamura, Kazuya Sato, Yusuke Mizukami, Yoshihiro Torimoto, Toshikatsu Okumura

**Affiliations:** a Department of Medicine, Division of Gastroenterology and Hematology/Oncology, Asahikawa Medical University; b Department of Hematology/Oncology, Asahikawa-Kosei General Hospital; c Oncology Center, Asahikawa Medical University Hospital, Asahikawa, Japan.

**Keywords:** acquired hemophilia A, Epstein–Barr virus-associated lymphoproliferative disease, lymphoproliferative disease

## Abstract

**Introduction:**

Acquired hemophilia A (AHA) is a rare bleeding disorder caused by autoantibodies against factor VIII (FVIII). Hematological malignancies, especially lymphoid malignancies, are known to be underlying causes of AHA; however, thus far, there is no report of AHA associated with Epstein–Barr-virus-associated T/natural killer-cell lymphoproliferative disease (EBV-T/NK-LPD). Here, we present a case of AHA that developed during treatment for EBV-T/NK-LPD.

**History:**

A 69-year-old man visited our hospital because of general fatigue. Blood examination showed pancytopenia, and computed tomography revealed whole-body lymphadenopathy, but there were no findings indicating hematological malignancy from bone marrow aspiration and cervical lymph node biopsy. The level of EBV DNA in peripheral blood was extremely high, and he was diagnosed with EBV-T/NK-LPD. EBV-T/NK-LPD improved with prednisolone (PSL) administration. Seventeen months after starting treatment, the patient complained of back and right leg pain. At that time, he had been treated with low-dose PSL, and EBV-T/NK-LPD was well controlled. Imaging revealed hematoma of the right iliopsoas muscle. Prolonged activated partial thromboplastin time (APTT) was the only abnormal finding in a screening coagulation test. FVIII coagulant activity was below detection limit, and FVIII inhibitor level was increased. From these results, he was diagnosed with AHA.

A higher dose of PSL was administered, and, after 1 month of treatment, FVIII activity gradually increased, and FVIII inhibitor level became undetectable. APTT also normalized, and complete remission was achieved and maintained for 13 months with low-dose PSL. During treatment, EBV-T/NK-LPD was well controlled.

**Conclusion:**

It is speculated that proliferating lymphocytes interfere with normal immune functions and that abnormal autoantibodies are produced from those lymphocytes in patients with LPD. Therefore, we speculate that EBV-infected and proliferating monoclonal NK cells might have modulated the immune system and produced autoantibodies against FVIII, thus causing AHA in this patient with EBV-T/NK-LPD.

## 1 Introduction

Acquired hemophilia A (AHA) is a rare bleeding disorder caused by autoantibodies against factor VIII (FVIII).^[1,2]^ AHA can be a cause of severe bleeding, resulting in high morbidity and mortality.^[1]^ Most AHA is idiopathic and may be also associated with malignancy, autoimmune diseases, and medication.^[2]^ Hematological malignancies, especially lymphoid malignancies, are known to be underlying causes of AHA^[1]^; however, thus far, there is no report of AHA associated with Epstein–Barr-virus-associated T/natural killer-cell lymphoproliferative disease (EBV-T/NK-LPD). Here, we present a case of AHA that developed during treatment for EBV-T/NK-LPD.

## 2 Case report

A 69-year-old man, who had been taking medication for diabetes, hypertension, and chronic kidney disease, visited our hospital because of general fatigue. Blood examination showed pancytopenia, and a computed tomography (CT) scan revealed whole-body lymphadenopathy. Bone marrow aspiration was normal without the findings of hemophagocytosis, and cervical lymph node biopsy showed no evidence of lymphoma or metastatic tumors. The titers of serum antibodies against EBV were as follows: EBV-viral capsid antigen antibody (EBV-VCA) IgG was 80 times, EBV-VCA IgM was <10 times, EBV-VCA IgA was 10 times, and EBV nuclear antigen (EBNA) was 20 times. We considered that these results were atypical because EBV-VCA IgA was slightly positive and EBNA was weakly positive. We calculated the level of EBV DNA in peripheral blood, and it was extremely high (38,000 copies/10^6^ cells). The monoclonality of EBV in peripheral blood was confirmed by Southern blotting analysis. To clarify which cells were infected with EBV, we separated B cells, T cells, and NK cells by flow cytometry and quantified the amount of EBV DNA in each fraction.^[3]^ Significant amounts of EBV DNA were detected in the NK cell fraction. We speculated that NK cells were infected with EBV and monoclonal NK cell proliferation, and we eventually diagnosed as EBV-T/NK-LPD (chronic active EBV infection of NK-cell type, systemic form according to WHO classification 2017). At this point, the coagulation tests were all within the normal range. The patient was treated with a high dose of prednisolone (PSL; 1 mg/kg/day). The symptoms were significantly improved, and pancytopenia was resolved, with normal hemoglobin level. The level of EBV DNA in peripheral blood was decreased and PSL dose was tapered gradually.

**Figure 1:**
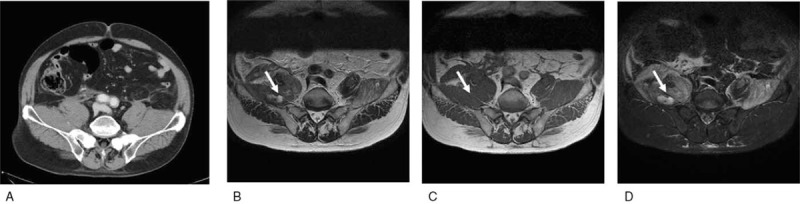
Imaging findings at admission. Enhanced computed tomography (A) showed swelling of the right iliopsoas and iliacus muscles. Magnetic resonance imaging showed an area (white arrows) in the right iliopsoas muscle that had a high signal on T2-weighted images (B), isointense signal on T1-weighted images (C), and without suppression on fat-suppression images (D), from these findings, the patient was diagnosed with hematoma of the right iliopsoas muscle.

**Figure 2:**
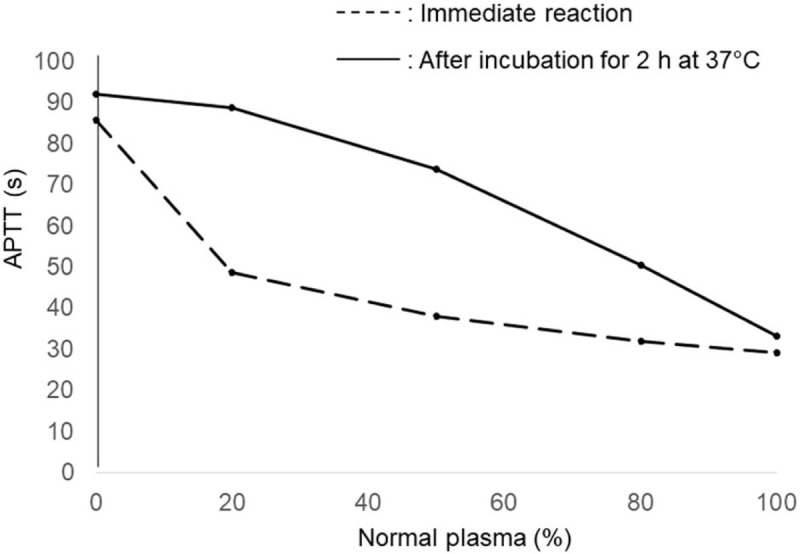
Result of mixing test. Activated partial thromboplastin time (APTT) was not corrected by normal plasma, which was clearer after incubation for 2 hours at 37°C than after immediate reaction.

**Figure 3:**
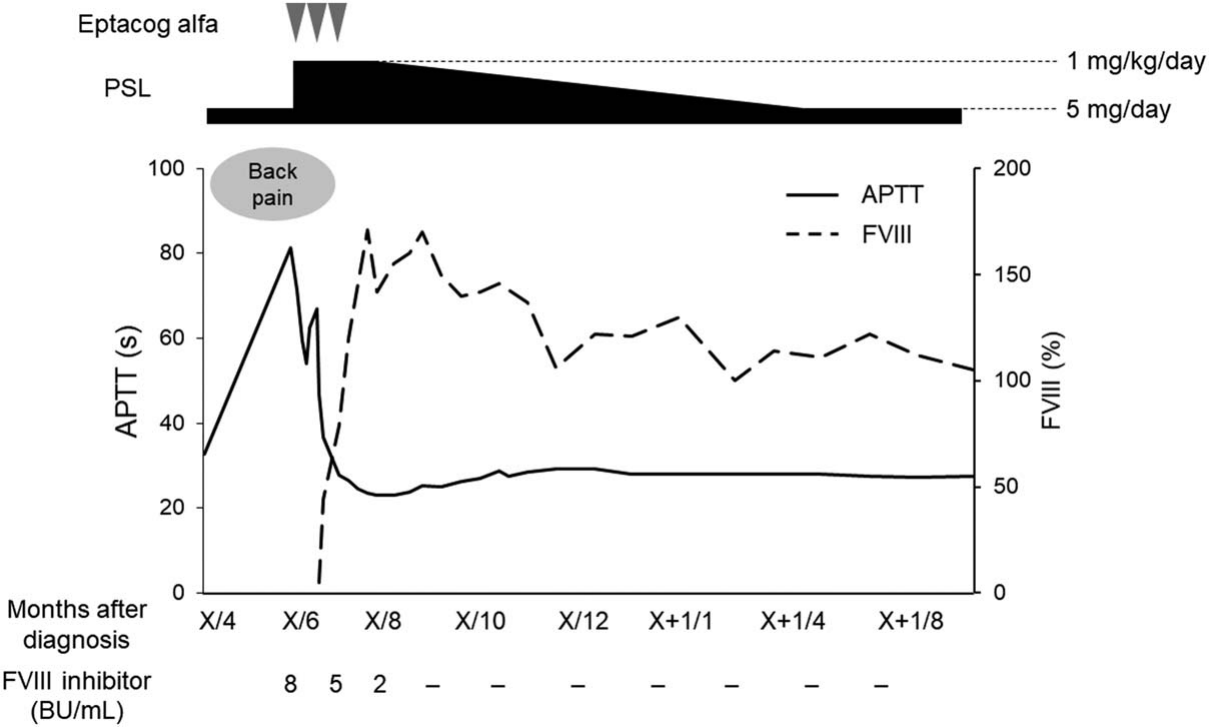
Clinical course from the diagnosis of acquired hemophilia A (AHA). Following diagnosis of AHA, a higher dose of prednisolone (PSL; 1 mg/kg/day) was administered, and eptacog alfa was administered for bleeding. After 1 month of these treatments, factor VIII (FVIII) activity increased gradually, and FVIII inhibitor level became undetectable. Activated partial thromboplastin time (APTT) was also normalized. PSL dose was gradually reduced, and AHA was maintained in complete remission with low-dose PSL (5 mg/day).

Seventeen months after starting treatment, the patient complained of back and right leg pain. At that time, he had been treated with low-dose PSL (5 mg/day), and EBV-T/NK-LPD was well controlled. Blood examination showed anemia without thrombocytopenia and a slight increase in inflammatory reaction (Table 1). CT and magnetic resonance imaging (MRI) were performed to find the causes of the pain, and revealed hematoma of the right iliopsoas muscle (Fig. 1). The coagulation test showed prolonged activated partial thromboplastin time (APTT). International normalized ratio of prothrombin time, plasma fibrinogen, and D-dimer were within the normal ranges (Table 1). The mixing test showed that APTT was not corrected by normal plasma (Fig. 2). FVIII coagulation activity was below the detection limit, and FVIII inhibitor level was increased. Factor V, VII and X activities were within the normal ranges (Table 1). On the basis of these results, the patient was diagnosed with AHA. Antinuclear, anti-double-stranded DNA, and antiphospholipid antibodies were all negative (Table 1). Internal malignancies were not detected by CT and MRI. A higher dose of PSL (1 mg/kg/day) was administered, and bleeding was treated with eptacog alfa, recombinant activated factor VII (rFVII), and fresh frozen plasma (FFP) transfusion. After 1 month of these treatments, FVIII activity was gradually increased, and FVIII inhibitor level became undetectable. APTT was also normalized with increasing FVIII. Further eptacog alfa treatment and FFP transfusion were not required, and complete remission (CR) was achieved, which was maintained for 13 months with low-dose PSL (5 mg/day) (Fig. 3). During treatment, the EBV DNA load was not increased, indicating that EBV-T/NK-LPD was well controlled.

**Table 1:** Laboratory data on admission.

WBC	13,000	/μL	TP	6.1	g/dL	PT-%	93	%	EBV DNA	220	copies/10^6^cells
Neu	85.8	%	Alb	3.3	g/dL	PT-INR	1.04				
Lym	9	%	T-bil	1.2	mg/dL	APTT	81.3	sec	HBsAg	0	IU/mL
Mono	4.7	%	D-bil	0.1	mg/dL	Fib	398	mg/dL	HBsAb	517.2	mIU/mL
Eos	0.4	%	ALP	202	IU/L	D-dimer	1.8	mg/dL	HBcAb	6.2	
Baso	0.1	%	AST	20	IU/L				HBV DNA	(–)	
RBC	204 × 10^4^	/μL	ALT	15	IU/L	FVIII	<1	%			
Hb	6.6	g/dl	LDH	236	IU/L	FVIII inhibitor	8	BU/mL	Antinucleotide antibody	(–)	
Ht	19.8	%	γ-GTP	15	IU/L	FV	86	%	anti-dsDNA antibody	(–)	
MCV	97.1	fL	BUN	33.8	mg/dL	FVII	132	%	antiphospholipid antibody	(–)	
MCH	32.4	pg	Cre	1.32	mg/dL	FX	88	%			
MCHC	33.3	%	Na	131	mg/dL						
Plt	24.5 × 10^4^	/μL	K	4.9	mg/dL						
			Cl	95	mg/dL						
			Ca	8.3	mg/dL						
			CRP	2.09	mg/dL						

## 3 Discussion

AH is a rare bleeding disorder. The incidence in the general population was estimated at 1.5 cases per million persons/year.^[1]^ AH is caused by circulating autoantibodies directed against a specific clotting factor.^[1]^ AHA caused by FVIII inhibitors is the most commonly reported type of AH.^[1]^ AHA increases with age, and the median age of patients at diagnosis was 64 to 78 years in the past report.^[2]^ Although approximately half of cases of AHA are idiopathic, AHA is also associated with autoimmune diseases, infections, medications, and internal malignancies.^[1,2]^ AHA occasionally develops after hematological malignancies such as LPDs^[4,5]^; however, there is no report of AHA associated with EBV-T/NK-LPD as in the present case.

The bleeding pattern of AHA is different from that of congenital hemophilia A. Most patients with congenital hemophilia A suffer from hemorrhages in the joints. In contrast, the majority of AHA patients have hemorrhages in the skin, muscles, soft tissue, and mucous membranes.^[1,2]^ In the current case, the patient had iliopsoas muscle hemorrhage. Therefore, AHA patients have a significant risk of developing a severe bleeding disorder associated with high morbidity and mortality rates. A previous study demonstrated that severe and life-threatening bleeding occurred in 70% to 90% of AHA patients, and the fatality rate was 5% to 10%.^[1]^ Hemostatic agents such as bypassing agents, rFVIIa, and activated prothrombin complex concentrates are considered as first-line treatment for bleeding episodes in AHA patients.^[1]^ In patients with lower titers of FVIII inhibitor (<5 BU/ml), human FVIII replacement and desmopressin might be adequate. Still, these treatments are deemed to be less successful in controlling bleeding.^[2]^ Our patient was successful in controlling bleeding by using rFVIIa, and we used FFP transfusion to support hemostatic agents.

The most common therapy for FVIII inhibitor eradication in AHA is steroids, alone (PSL 1–2 mg/kg/day for 4–6 weeks) or in combination with cyclophosphamide (1–2 mg/kg/day for a maximum of 5 weeks).^[1,2]^ These therapies can achieve CR in 70% to 80% of AHA patients.^[1]^ In the current case, the patient could be treated by PSL alone.

Previous studies have demonstrated that various types of malignancies can promote onset of AHA.^[1,2,4]^ Prostate and lung cancer seem to be the most common among solid tumors,^[4]^ while LPDs (lymphoma, chronic lymphocytic leukemia, plasma cell dyscrasias, or mycosis fungoides) are the most predominant among hematological malignancies.^[4,5]^

EBV-T/NK-LPD is one of the LPDs. EBV is a linear, double-stranded DNA virus that can cause both acute and chronic infections. The primary symptoms of EBV-T/NK-LPD are fever, hepatosplenomegaly, lymphadenopathy, and liver dysfunction, which are similar to the symptoms of infectious mononucleosis.^[5]^ Patients with EBV-T/NK-LPD often need intensive treatments such as chemotherapy and hematopoietic stem cell transplantation because of high morbidity and mortality.^[3]^ The cause of EBV-T/NK-LPD is clonal systemic proliferation of EBV-infected cytotoxic T or NK cells, but the precise mechanisms of the relationship between EBV infection and LPD are not clear.^[3,6]^ The host immune system can remove EBV-infected T and NK cells under normal immune conditions. However, some particular immunocompromised states, such as aging, drug treatment, or malignancy, may induce insufficient eradication of the EBV-infected T and NK cells.^[5]^ The increase in EBV-infected cells leads to an increase in EBV DNA in peripheral blood, which is an essential parameter for evaluation of EBV-T/NK-LPD.^[5]^ Although we could not define the conclusive causes of EBV-T/NK-LPD in our patient, aging, diabetes, and chronic kidney disease might have contributed to this virus-associated LPD.

LPDs are known to be associated with various autoimmune diseases, including AHA.^[4,5,7–9]^ It is speculated that proliferating lymphocytes interfere with normal immune functions, and abnormal autoantibodies are produced by those lymphocytes in patients with LPDs. EBV-T/NK-LPD is one of the LPDs; however, there are no reports of it being related to autoimmune diseases, including AHA. On the basis of the present case, we speculate that EBV-infected and proliferating monoclonal NK cells might modulate the immune system and produce autoantibodies against FVIII and cause AHA. In contrast, chronic systemic inflammation, including viral infections, may cause AHA.^[1,2]^ Hepatitis C virus and human immunodeficiency virus are reported to be associated with AHA,^[10,11]^ whereas the association with EBV infection has not been reported previously.

## 4 Conclusion

To the best of our knowledge, we present the first case of AHA associated with EBV-T/NK-LPD. We speculate that EBV-infected and proliferating monoclonal NK cells may have modulated the patient immune system and produced autoantibodies against FVIII, which eventually induced AHA in this EBV-T/NK-LPD patient.

## Acknowledgments

We thank Cathel Kerr, BSc, PhD, from Edanz Group (https://en-author-services.edanz.com/ac) for editing a draft of this manuscript.

## Author contributions

**Data curation:** Masayo Yamamoto, Chihiro Sumi, Sho Igarashi, Takeshi Saito, Nodoka Tsukada, Yasumichi Toki, Mayumi Hatayama, Junki Inamura, Kazuya Sato.

**Investigation:** Masayo Yamamoto, Motohiro Shindo, Chihiro Sumi, Sho Igarashi, Takeshi Saito, Nodoka Tsukada, Yasumichi Toki, Mayumi Hatayama, Junki Inamura, Kazuya Sato, Yusuke Mizukami, Yoshihiro Torimoto, Toshikatsu Okumura.

**Writing – original draft:** Masayo Yamamoto.

**Writing – review & editing:** Motohiro Shindo, Yusuke Mizukami, Yoshihiro Torimoto, Toshikatsu Okumura.
